# Unveiling Targets for Treating Postoperative Pain: The Role of the TNF-α/p38 MAPK/NF-κB/Nav1.8 and Nav1.9 Pathways in the Mouse Model of Incisional Pain

**DOI:** 10.3390/ijms231911630

**Published:** 2022-10-01

**Authors:** Flávia Oliveira de Lima, Pedro Santana Sales Lauria, Renan Fernandes do Espírito-Santo, Afrânio Ferreira Evangelista, Tâmara Magalhães Oliveira Nogueira, Dionéia Araldi, Milena Botelho Pereira Soares, Cristiane Flora Villarreal

**Affiliations:** 1 Health Department, State University of Feira de Santana, Feira de Santana 44036900, BA, Brazil; 2 School of Pharmacy, Federal University of Bahia, Salvador 40170115, BA, Brazil; 3 Gonçalo Moniz Institute, Oswaldo Cruz Foundation, Salvador 40296710, BA, Brazil; 4 SENAI Institute of Innovation in Advanced Health Systems, University Center SENAI/CIMATEC, Salvador 41650010, BA, Brazil; 5 Department of Structural and Functional Biology, Institute of Biology, University of Campinas, Campinas 13083-862, SP, Brazil

**Keywords:** postoperative pain, p38 MAPK, NF-κB, TNF-α, voltage-gated sodium channels

## Abstract

Although the mouse model of incisional pain is broadly used, the mechanisms underlying plantar incision-induced nociception are not fully understood. This work investigates the role of Na_v_1.8 and Na_v_1.9 sodium channels in nociceptive sensitization following plantar incision in mice and the signaling pathway modulating these channels. A surgical incision was made in the plantar hind paw of male Swiss mice. Nociceptive thresholds were assessed by von Frey filaments. Gene expression of *Na_v_1.8*, *Na_v_1.9*, *TNF-α*, and *COX-2* was evaluated by Real-Time PCR in dorsal root ganglia (DRG). Knockdown mice for Na_v_1.8 and Na_v_1.9 were produced by antisense oligodeoxynucleotides intrathecal treatments. Local levels of TNF-α and PGE_2_ were immunoenzymatically determined. Incised mice exhibited hypernociception and upregulated expression of *Na_v_1.8* and *Na_v_1.9* in DRG. Antisense oligodeoxynucleotides reduced hypernociception and downregulated *Na_v_1.8* and *Na_v_1.9*. TNF-α and *COX-2*/PGE_2_ were upregulated in DRG and plantar skin. Inhibition of TNF-α and COX-2 reduced hypernociception, but only TNF-α inhibition downregulated *Na_v_1.8* and *Na_v_1.9*. Antagonizing NF-κB and p38 mitogen-activated protein kinase (MAPK), but not ERK or JNK, reduced both hypernociception and hyperexpression of *Na_v_1.8* and *Na_v_1.9*. This study proposes the contribution of the TNF-α/p38/NF-κB/Na_v_1.8 and Na_v_1.9 pathways to the pathophysiology of the mouse model of incisional pain.

## 1. Introduction

Acute pain commonly affects patients who undergo surgical procedures. Although this is an expected physiological response to mechanical injury, postoperative pain becomes persistent in 10–50% of patients after common operations [1]. Chronification of postoperative pain complicates recovery from surgery, lengthens hospitalization time, and increases morbidity [2,3]. Transitioning from acute to chronic pain is marked by long-lasting functional alterations in nociceptive pathways following intense or repetitive painful stimuli [4]. In fact, there is a positive correlation between the intensity of acute postoperative pain and pain chronification [5]. Hence, understanding the pathophysiology of postoperative pain could disclose novel druggable molecular targets for a more effective treatment of acute pain and for avoiding postoperative pain chronification.

Animal models have been developed for studying the pathophysiological mechanisms of postoperative pain as well as the effects of pharmacological treatments. The preclinical model of incisional pain in rats was introduced by Brennan et al. [3] and later adapted to mice by Pogatzki and Raja [6]. This model simulates postoperative pain in humans and consists in making a surgical incision in the forepaw of the anesthetized mouse. Rodents submitted to paw incision develop nociceptive sensitization to both mechanical and thermal stimuli [7] as well as changes in the electrophysiological properties of afferent fibers, including decreased activation thresholds and increased receptive fields, leading to non-evoked pain-related behaviors, primary hyperalgesia, and secondary hyperalgesia [8,9,10]. Although the mouse model of incisional pain has allowed researchers to characterize the pain-like behaviors, such as electrophysiological changes in the nociceptors and some pathophysiological mediators relevant to postoperative nociception [6,8,11], the molecular mechanisms underlying the pronociceptive state induced by plantar incision remain not fully understood.

Different types of pain are associated with distinctive pathophysiological mechanisms. Likewise, different murine models of pain promote diverse neurochemical alterations in the nervous system [12]. The mouse model of incisional pain also has its own peculiarities; for instance, it is not the afferent barrage induced by the incision that causes nociceptive sensitization, but rather the persistent inputs from the wound [13]. Ongoing painful inputs are associated with changes in the pattern of gene expression in cells that make up the pain modulatory pathways, favoring the perpetuation of a pronociceptive state [14]. These changes involve the upregulation of hypernociceptive mediators and transmembrane channels, such as voltage-gated sodium channels (VGSCs), responsible for the electric activity and the conduction of action potentials in nociceptive primary afferents.

VGSCs can be classified based on their sensitivity to tetrodotoxin, a neurotoxin present in pufferfish and other organisms. Na_v_1.8 and Na_v_1.9 are tetrodotoxin-resistant (TTX-R) VGSCs expressed in nociceptive sensory neurons that are important for the conduction of harmful stimuli [15]. It has been suggested that TTX-R VGSCs are particularly relevant in the development of inflammatory and painful neuropathic conditions [16,17,18]. Ma et al. [19] showed increased TTX-R currents in the dorsal root ganglia of rats submitted to paw incision, suggesting a role for Na_v_1.8 and Na_v_1.9 in the pathophysiology of incisional pain. The increase in TTX-R currents can be driven by several mechanisms, including sensitization by hypernociceptive mediators and/or upregulation by transcription factors.

NF-κB is a major transcription factor associated with the production of hypernociceptive mediators [20] and the modulation of VGSCs [21,22]. Han et al. [21], using a skin/muscle incision and retraction model of postoperative pain, showed that the NF-κB signaling pathway upregulates the expression of *Na_v_1.7* in the dorsal root ganglia of rats. The NF-κB pathway can be regulated by mitogen-activated protein kinases (MAPK), which are involved in several cellular processes, including the modulation of painful states [23]. Pogatzki-Zahn et al. [9] reported changes in gene expression in the dorsal root ganglia of mice submitted to the model of incisional pain by using mass spectrometry-based proteomics, showing the regulation of 44 proteins, including many associated with immune and inflammatory responses, the regulation of cytokines production, and the modulation of MAPK cascades.

Although a role for VGSCs in incisional pain has been suggested, the molecular pathways leading to their activation/sensitization are not clear. Understanding these pathways will allow a deeper comprehension of incisional pain and potentially bring to light new approaches for its treatment. Therefore, considering the relevance of TTX-R VGSCs for the establishment of pathological pain states and the evidence suggesting the activation of MAPK cascades following plantar incision, this study aims to investigate the contribution of Na_v_1.8 and Na_v_1.9 to paw incision-induced hypernociception as well as the contribution of the MAPK/NF-κB signaling pathway in the modulation of these channels in the dorsal root ganglia (DRG).

## 2. Results

### 2.1. Plantar Incision-Induced Mechanical Hypernociception Accompanies Increased Expression of Na_v_1.8 and Na_v_1.9 in the Dorsal Root Ganglia

**Figure 1:**
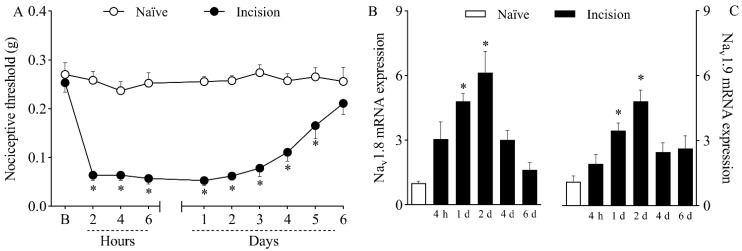
Mechanical hypernociception and increased expression of *Na_v_1.8* and *Na_v_1.9* in the dorsal root ganglia (DRG) caused by plantar incision. Mice were induced to the plantar incision model of postoperative pain. No surgical procedures were performed on *naïve* mice. (**A**) The mechanical nociceptive threshold (axis of ordinates) was evaluated at baseline (**B**) and at different times in hours and days after plantar incision (axis of abscissas). Values represent the filament weight (g) to which mice respond in 50% of presentations. Data are expressed as means ± SD (*n* = 6). * *p* < 0.05 compared with *naïve* group. Two-way ANOVA followed by Bonferroni’s test. (**B**,**C**) The levels of Na_v_1.8 (**B**) and Na_v_1.9 (**C**) mRNA in the DRG (axis of ordinates) were determined by Real-Time PCR at different times in hours (h) and days (d) after plantar incision (axis of abscissas). Target gene expression (2^−ΔΔCt^) was determined using *Gapdh* as housekeeping gene. Data are expressed as means ± SD (*n* = 6). * *p* < 0.05 compared with naïve group. One-way ANOVA followed by Tukey’s test.

Mice submitted to plantar incision developed nociceptive sensitization, as shown by the decrease in nociceptive thresholds depicted in Figure 1A. While naïve mice exhibited a constant response to mechanical stimuli throughout the experiment, surgically manipulated mice presented significantly lower mechanical thresholds (*p* < 0.05) from two hours to five days after plantar incision, recovering in the sixth day.

Real-Time PCR analyses revealed that plantar incision increased (*p* < 0.05) both Na_v_1.8 mRNA (Figure 1B) and Na_v_1.9 mRNA (Figure 1C) expression in the dorsal root ganglia of mice on the first and second days after surgery. Transcriptional activity peaked on day two, with a sixfold increase in Na_v_1.8 mRNA and a fourfold increase in Na_v_1.9 mRNA compared to the transcriptional levels of naïve mice.

### 2.2. Downregulating Na_v_1.8 and Na_v_1.9 in the Dorsal Root Ganglia Reduces Plantar Incision-Induced Hypernociception

**Figure 2:**
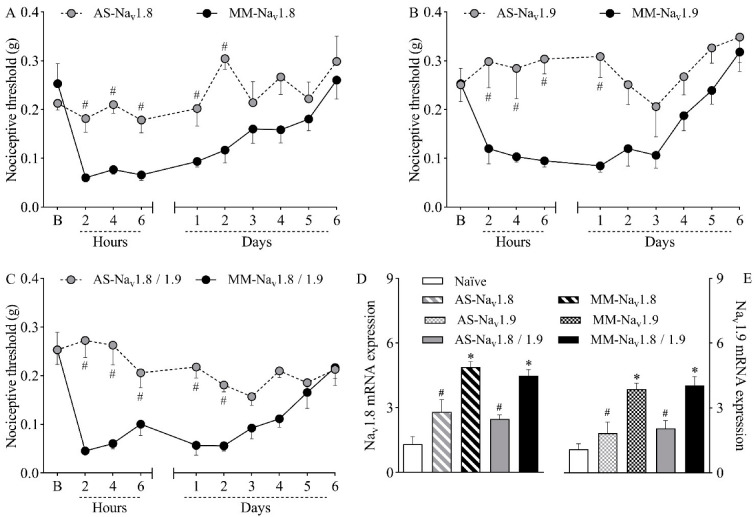
Mice were treated intrathecally with antisense oligodeoxynucleotide targeting Na_v_1.8 (AS-Na_v_1.8), Na_v_1.9 (AS-Na_v_1.9), both antisense treatments combined (AS-Na_v_1.8/1.9), mismatch oligodeoxynucleotide targeting Na_v_1.8 (MM-Na_v_1.8), Na_v_1.9 (MM-Na_v_1.9), or both mismatches combined (MM-Na_v_1.8/1.9). Mice were treated once a day (10 µg in 5 µL) for four days (final dose of 40 µg) and then induced to the plantar incision model of postoperative pain one hour after the last treatment. No treatments or surgical procedures were performed on naïve mice. (**A**–**C**) The mechanical nociceptive threshold (axis of ordinates) was evaluated at baseline (**B**) and at different times in hours and days after plantar incision (axis of abscissas). Values represent the filament weight (g) to which mice respond in 50% of presentations. Data are expressed as means ± SD (*n* = 6). # *p* < 0.05 compared with the respective mismatch group. Two-way ANOVA followed by Bonferroni’s test. (**D**,**E**) The levels of Na_v_1.8 and Na_v_1.9 mRNA in the DRG (axis of ordinates) were determined by Real-Time PCR two days after plantar incision. Target gene expression (2^−ΔΔCt^) was determined using *Gapdh* as housekeeping gene. Data are expressed as means ± SD (*n* = 6). * *p* < 0.05 compared with naïve group. # *p* < 0.05 compared with the respective mismatch group. One-way ANOVA followed by Tukey’s test.

To investigate the causal relationship between the increased expression of Na_v_1.8 and Na_v_1.9 channels and nociceptive sensitization, mice were intrathecally treated with antisense oligodeoxynucleotides (ODN) targeting Na_v_1.8 (Figure 2A), Na_v_1.9 (Figure 2B), or both sodium channels (Figure 2C) prior to plantar incision. Within-subject comparisons showed no significant differences between thresholds relative to baseline at any time point for groups treated with antisense ODN for Na_v_1.8 or Na_v_1.9, indicating that antisense-treated mice did not develop incision-induced hypernociception. On the other hand, within-subject differences relative to baseline were observed for up to two days in groups treated with mismatch ODN for Na_v_1.8 or Na_v_1.9 (*p* < 0.05), after which mechanical thresholds returned to baseline levels, suggesting reversal of the hypernociception. Importantly, while hypernociception persisted, all antisense treatments resulted in significantly higher nociceptive thresholds when compared to the corresponding mismatch groups (*p* < 0.05). Antisense ODN treatment for Na_v_1.8 was significantly different from the mismatch group for up to two days after surgery (Figure 2A; *p* < 0.05), whereas antisense ODN treatment for reducing Na_v_1.9 expression was significantly different from the mismatch group for up to one day after surgery (Figure 2B; *p* < 0.05). When hyperexpressed, Na_v_1.8 and Na_v_1.9 mRNA were simultaneously downregulated by the combined treatment with antisense ODN, and statistical differences between groups were observed for up to two days after surgery (Figure 2C, *p* < 0.05).

Ral Time PCR analyses were performed at the second post-operative day, when the hyperexpression of the TTX-R VGSC peaked, as shown in Figure 1B,C. The analyses confirmed that the treatment with antisense ODN effectively reduced the expression of both sodium channels, validating the results from the nociception test. While mismatch-treated mice showed increased expression of *Na_v_1.8* (Figure 2D; *p* < 0.05) and *Na_v_1.9* (Figure 2E; *p* < 0.05) compared to naïve mice, mRNA levels were significantly lower in both antisense groups compared to their corresponding mismatches (*p* < 0.05). The simultaneous treatment with both antisense ODNs reduced the mRNA expression of both sodium channels (Figure 2D,E; *p* < 0.05). This result agrees with the abolishment of mechanical hypernociception promoted by the combined treatment.

### 2.3. Plantar Incision Leads to Increased Expression of the Hypernociceptive Mediators TNF-α and COX-2/PGE_2_ in the Dorsal Root Ganglia and in the Plantar Skin

**Figure 3:**
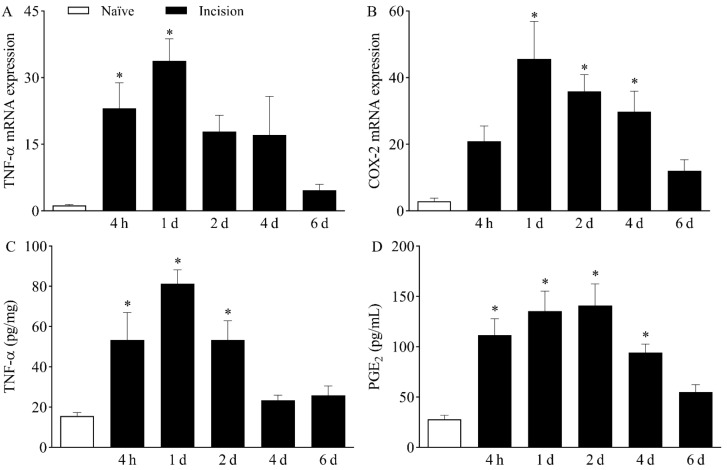
Increased expression of TNF-α and *COX-2*/PGE_2_ following plantar incision. Mice were induced to the plantar incision model of postoperative pain. No surgical procedures were performed on naïve mice. (**A**,**B**) The levels of TNF-α mRNA (**A**) and COX-2 mRNA (**B**) in the DRG (axis of ordinates) were determined by Real-Time PCR at different times in hours (h) and days (d) after plantar incision (axis of abscissas). Target gene expression (2^−ΔΔCt^) was determined using *Gapdh* as housekeeping gene. Data are expressed as means ± SD (*n* = 6). * *p* < 0.05 compared with naïve group. One-way ANOVA followed by Tukey’s test. (**C**,**D**) The levels of TNF-α (**C**) and PGE_2_ (**D**) in the plantar skin of the incised paw (axis of ordinates) were quantified at different times in hours (h) and days (d) after plantar incision (axis of abscissas). Data are expressed as means ± SD (*n* = 6). * *p* < 0.05 compared with naïve group. One-way ANOVA followed by Tukey’s test.

After determining that TTX-R VGSCs play a role in plantar incision-induced mechanical hypernociception, the local presence of hypernociceptive mediators that modulate the expression and activity of Na_v_1.8 and Na_v_1.9 was investigated. The expression of TNF-α mRNA (Figure 3A) was increased in the DRG at four hours and one day after plantar incision, peaking at day one, when compared to naïve mice (*p* < 0.05). Likewise, the expression of COX-2 mRNA (Figure 3B) increased in the DRG from day one to day four following surgery, also peaking at day one, in comparison with *naïve* mice (*p* < 0.05).

The upregulation of the above-mentioned genes in the DRG was associated with increased plantar skin levels of both TNF-α (Figure 3C) and PGE_2_ (Figure 3D) in the surgically manipulated paw when compared to *naïve* mice (*p* < 0.05). TNF-α levels were significantly higher from four hours to two days after incision, while PGE_2_ levels were increased from four hours to up to four days following plantar incision.

### 2.4. TNF-α and Prostanoids Contribute to Hypernociception, but Only TNF-α Contributes to Upregulation of Na_v_1.8 and Na_v_1.9 in the Dorsal Root Ganglia

**Figure 4:**
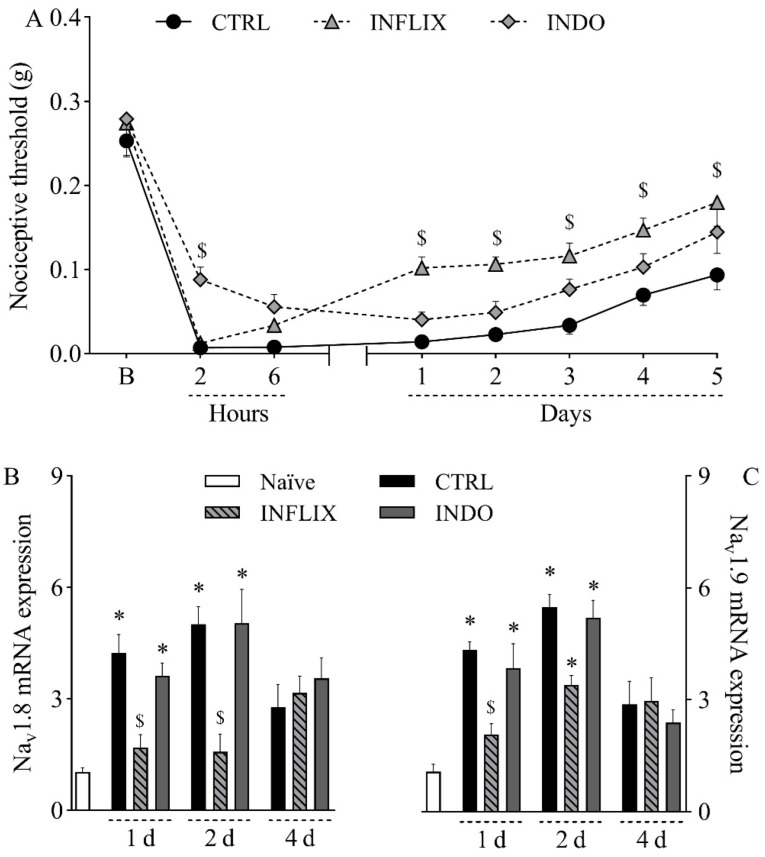
Contribution of TNF-α and prostanoids to mechanical hypernociception and upregulation of *Na_v_1.8* and *Na_v_1.9* in the dorsal root ganglia following plantar incision. Mice were treated with the anti-TNF-α monoclonal antibody infliximab (INFLIX; 10 mg/kg, intraperitoneally), the non-selective COX inhibitor indomethacin (INDO; 10 mg/kg, orally), or the vehicle (CTRL; control group). Plantar incision model of postoperative pain was induced one hour later. No surgical procedures nor treatments were performed on naïve mice. (**A**) The mechanical nociceptive threshold (axis of ordinates) was evaluated at baseline (**B**) and at different times in hours and days after plantar incision (axis of abscissas). Values represent the filament weight (g) to which mice respond in 50% of presentations. Data are expressed as means ± SD (*n* = 6). $ *p* < 0.05 compared with CTRL group. Two-way ANOVA followed by Bonferroni’s test. (**B**,**C**) The levels of Na_v_1.8 mRNA (**B**) and Na_v_1.9 mRNA (**C**) in the DRG (axis of ordinates) were determined by Real-Time PCR at different times in days (d) after plantar incision (axis of abscissas). Target gene expression (2^−ΔΔCt^) was determined using *Gapdh* as housekeeping gene. Data are expressed as means ± SD (*n* = 6). * *p* < 0.05 compared with naïve group. $ *p* < 0.05 compared with CTRL group. One-way ANOVA followed by Tukey’s test.

Since both TNF-α and PGE_2_ were upregulated in the incised paw, the role of these hypernociceptive mediators in the pathophysiology of the mouse model of incisional pain was next investigated by pharmacological assays. The treatment with the anti-TNF-α monoclonal antibody infliximab (10 mg/kg, intraperitoneally) reduced mechanical hypernociception from the first to the fifth day after plantar incision (Figure 4A; *p* < 0.05). It also significantly reduced the upregulation of Na_v_1.8 mRNA (Figure 4B; *p* < 0.05) in the first and second days after surgery as well as Na_v_1.9 mRNA (Figure 4C; *p* < 0.05) in the first day after surgery. On the other hand, the treatment with the non-selective COX inhibitor indomethacin (10 mg/kg, orally) reduced mechanical hypernociception only at two hours after plantar incision (Figure 4A; *p* < 0.05) without interfering with the expression of *Na_v_1.8* (Figure 4B) or *Na_v_1.9* (Figure 4C) in the DRG.

### 2.5. The p38 MAPK/NF-κB Pathway Plays a Role in the Pathophysiology of the Mouse Model of Incisional Pain

**Figure 5:**
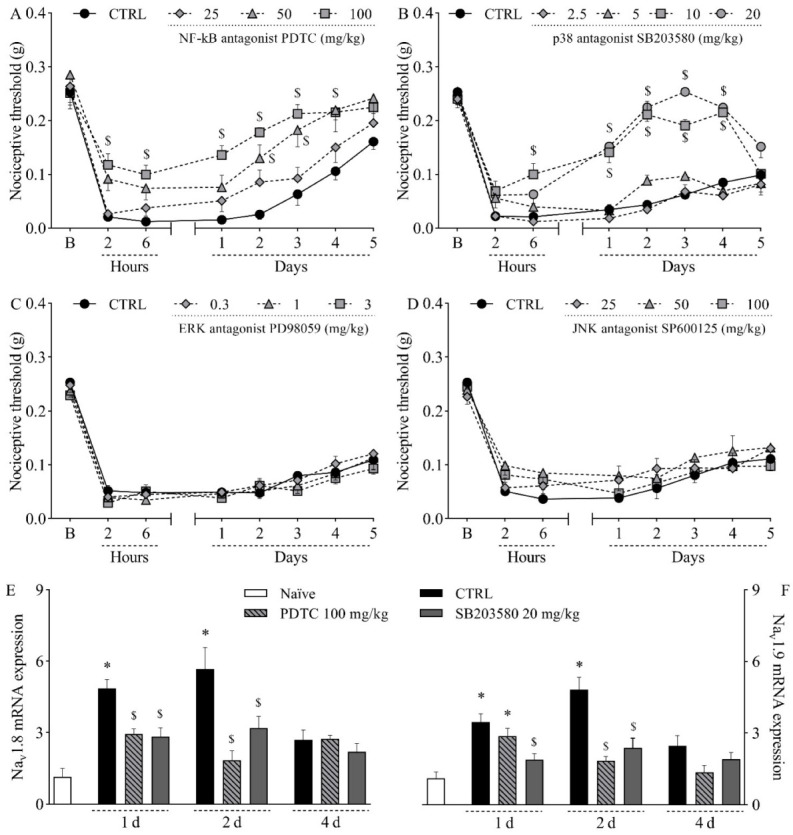
Influence of different MAPK and NF-κB antagonists on the mechanical hypernociception and on the upregulation of *Na_v_1.8* and *Na_v_1.9* in the dorsal root ganglia following plantar incision. Mice were treated with the NF-kB antagonist PDTC (25–100 mg/kg, intraperitoneally), the p38 antagonist SB203580 (2.5–20 mg/kg, intraperitoneally), the ERK antagonist PD98059 (0.3–3 mg/kg, subcutaneously), the JNK antagonist SP600125 (25–100 mg/kg, subcutaneously), or the vehicle (CTRL; control group) one hour before the induction of the plantar incision model of postoperative pain. No surgical procedures nor treatments were performed on the naïve mice. (**A**–**D**) The mechanical nociceptive threshold (axis of ordinates) was evaluated at baseline (**B**) and at different times in hours and days after plantar incision (axis of abscissas). Values represent the filament weight (g) to which mice respond in 50% of presentations. Data are expressed as means ± SD (*n* = 6). $ *p* < 0.05 compared with CTRL group. Two-way ANOVA followed by Bonferroni’s test. (**E**,**F**) The levels of Na_v_1.8 mRNA (**E**) and Na_v_1.9 mRNA (**F**) in the DRG (axis of ordinates) were determined by Real-Time PCR at different times in days (d) after plantar incision (axis of abscissas). Target gene expression (2^−ΔΔCt^) was determined using *Gapdh* as housekeeping gene. Data are expressed as means ± SD (*n* = 6). * *p* < 0.05 compared with naïve group. $ *p* < 0.05 compared with CTRL group. One-way ANOVA followed by Tukey’s test.

To further investigate the pathophysiology of the mouse model of incisional pain, the involvement of MAPK enzymes and the transcription factor NF-κB was evaluated by pharmacological assays. The intraperitoneal treatment with the NF-kB antagonist PDTC at 100 mg/kg significantly reduced mechanical hypernociception from two hours to four days after incision, as determined by comparison with the control group (Figure 5A; *p* < 0.05). It is noteworthy that the lack of statistical difference at five hours can be attributed to the return of mechanical thresholds in the control group to baseline values, since within-subject comparisons revealed no difference between thresholds at baseline and at five days in that group (*p* = 0.2958). PDTC at 50 mg/kg significantly reduced mechanical hypernociception at two hours and two days after plantar incision (Figure 5A; *p* < 0.05). PDTC at 25 mg/kg did not affect mechanical hypernociception (Figure 5A). Hypernociception was also reduced by the intraperitoneal treatment with the p38 antagonist SB203580 from the sixth hour to the fourth day post-incision at 10 mg/kg, and from the first to the fourth day post-incision at 20 mg/kg (Figure 5B; *p* < 0.05). SB203580 at 2.5 and 5 mg/kg did not affect mechanical hypernociception (Figure 5B). On the other hand, neither the ERK antagonist PD98059 (0.3–3 mg/kg, subcutaneously; Figure 5C) nor the JNK antagonist SP600125 (25–100 mg/kg, subcutaneously; Figure 5D) altered plantar incision-induced mechanical hypernociception. Both the NF-kB antagonist PDTC at 100 mg/kg and the p38 antagonist SB203580 at 20 mg/kg reduced the hyperexpression of *Na_v_1.8* (Figure 5E; *p* < 0.05) and *Na_v_1.9* (Figure 5F; *p* < 0.05) in the DRG.

### 2.6. Activating the NF-κB Pathway Increases the Expression of TNF-α and COX-2/PGE2 in the Dorsal Root Ganglia and in the Plantar Skin of Incised Mice

**Figure 6:**
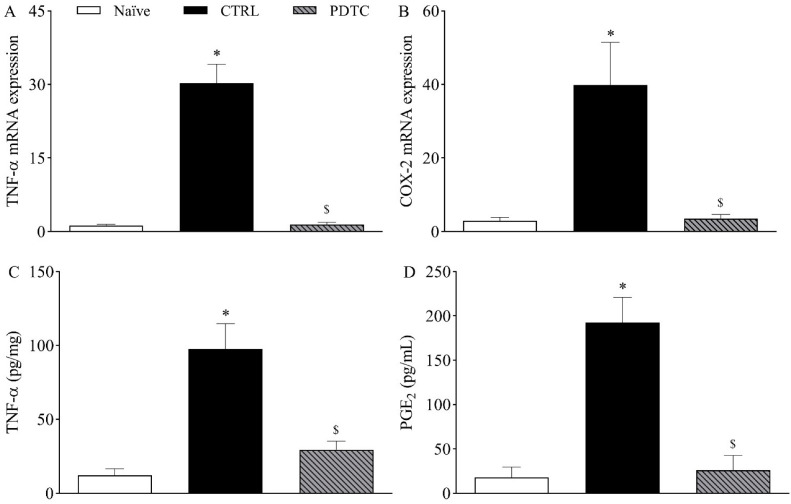
Influence of NF-κB on the expression of TNF-α and *COX-2*/PGE_2_ following plantar incision. Mice were treated with the NF-kB antagonist PDTC (100 mg/kg, intraperitoneally) or the vehicle (CTRL; control group) one hour before the induction of the plantar incision model of postoperative pain. No surgical procedures were performed on naïve mice. (**A**,**B**) The levels of TNF-α mRNA (**A**) and COX-2 mRNA (**B**) in the DRG (axis of ordinates) were determined by Real-Time PCR one day after plantar incision. Target gene expression (2^−ΔΔCt^) was determined using *Gapdh* as housekeeping gene. Data are expressed as means ± SD (*n* = 6). * *p* < 0.05 compared with naïve group. $ *p* < 0.05 compared with CTRL group. One-way ANOVA followed by Tukey’s test. (**C**,**D**) The levels of TNF-α (**C**) and PGE_2_ (**D**) in the plantar skin of the incised paw (axis of ordinates) were quantified one day after plantar incision. Data are expressed as means ± SD (*n* = 6). * *p* < 0.05 compared with naïve group. $ *p* < 0.05 compared with CTRL group. One-way ANOVA followed by Tukey’s test.

To determine whether TNF-α and *COX-2*/PGE_2_ were downstream targets of NF-κB in the mouse model of incisional pain, the expression of these hypernociceptive mediators following the pharmacological inhibition of NF-κB was investigated on the first day after plantar incision. Corroborating previous experiments, the expression of TNF-α mRNA (Figure 6A) and COX-2 mRNA (Figure 6B) in the DRG as well as the plantar skin levels of TNF-α (Figure 6C) and PGE_2_ (Figure 6D) following plantar incision were increased when compared to naïve mice (*p* < 0.05). The treatment with the NF-kB antagonist PDTC (100 mg/kg, intraperitoneally) significantly reduced the expression of TNF-α mRNA (Figure 6A) and COX-2 mRNA (Figure 6B) in the DRG and the plantar skin levels of TNF-α (Figure 6C) and PGE_2_ (Figure 6D) when compared to the control mice (*p* < 0.05).

## 3. Discussion

Coping with pain has been a constant in human history. As science evolved, so did our understanding of pain, which has gained new layers of complexity, currently including both physical and emotional features [24]. However, many aspects of the unpleasant experience of pain remain inscrutable. Persistent postoperative pain affects a significant number of individuals worldwide, although there is still not a concise answer to why this happens. This work contributes to the knowledge on postoperative pain by proposing a role for the TNF-α/p38 MAPK/NF-κB/Na_v_1.8 and Na_v_1.9 pathways in the pathophysiology of the mouse model of incisional pain. Our data suggest that these are potential pharmacological targets not yet addressed in the treatment of postoperative pain.

Mice submitted to the model of incisional pain developed nociceptive sensitization to mechanical stimuli, in agreement with previous reports by many authors who worked with this model [6,7,8,9]. The development of mechanical sensitization seems to be driven by early events, since a drastic drop in mechanical thresholds was registered two hours after plantar incision. Nevertheless, structural long-lasting alterations also seem to take place, as nociceptive sensitization lasted for up to five days. This phenomenon correlates with clinical symptoms of patients with postoperative pain, some of which develop hyperalgesia [25,26]. As a consequence, pain can be spontaneously evoked at rest and can be intensified by non-noxious stimuli and activities, such as mild touch, walking, and even coughing [27].

Pain processing involves multiple steps that are controlled by various intracellular signaling molecules, membrane receptors, and ion channels Na_v_1.8 and Na_v_1.9 are TTX-R VGSCs expressed in nociceptive neurons and are responsible for the conduction of thermal and mechanical noxious stimuli [15,28]. Experimental data have shown the intensification of TTX-R currents in painful pathological conditions, including inflammatory pain [29], spinal cord injury [30], and gouty arthritis [31]. In this study, both Na_v_1.8 and Na_v_1.9 mRNA were hyperexpressed in the DRG of incised mice, suggesting that their upregulation could be at least partially responsible for nociceptive sensitization. The upregulation of VGSCs alters the electrogenic properties of neurons, leading to increased excitability and chronification of pain [32]. This hypothesis was confirmed with the use of antisense ODN treatments, which established a clear connection between the selective downregulation of Na_v_1.8 and Na_v_1.9 mRNA and the reduction in plantar incision-induced hypernociception. The use of antisense ODN targeting TTX-R VGSCs has been shown to promote antinociceptive effects in murine models of pain, such as chronic orofacial hyperalgesia [33], acute and chronic inflammatory hypernociception [17,18], and social defeat stress-induced hyperalgesia [34]. In contrast with the results described herein, Joshi et al. [35] reported that treatment with antisense ODN targeting Na_v_1.8 had no effect on the mechanical allodynia induced by plantar incision in rats. These discrepant results may be due to the use of different animal species or to the variable effectiveness of different antisense ODN treatments in reducing the expression of their targets.

Sodium channels can be modulated by hypernociceptive mediators that ultimately increase neuronal depolarization and hence the transmission of painful stimuli. One of the most well-known molecules that facilitates pain transmission is PGE_2_, a major inflammatory mediator and product of the arachidonic acid pathway [36]. The hypernociceptive mechanism of PGE_2_ involves the activation of the protein kinase C (PKC) and the protein kinase A (PKA) pathways in nociceptive neurons after binding to EP1 and EP4 receptors, respectively. These kinases phosphorylate and modulate the function of various cellular components, including Na_v_1.8 and Na_v_1.9 sodium channels [36,37]. In the present study, plantar incision was followed by increased PGE_2_ levels in the plantar skin of mice until the fourth day. Since COX-2 mRNA was upregulated in the DRG, it is likely that this cyclooxygenase isoform contributed to plantar PGE_2_ synthesis. Corroborating this hypothesis, Ma et al. [19] have shown that selective inhibition of COX-2 by celecoxib inhibits mechanical hypernociception and reduces TTX-R currents in the DRG of rats submitted to plantar incision. These data strongly suggest a role for PGE_2_ in the sensitization of Na_v_1.8 and/or Na_v_1.9 in postoperative nociception. In the present work, prostaglandins, especially PGE_2_, seem to be particularly relevant to the early stages of nociceptive sensitization since pharmacological inhibition of COX by indomethacin partially prevented hypernociception at the second hour post-incision. This agrees with the kinetic profile of PGE_2_, which is quickly released in tissues after mechanical injuries [36]. On the other hand, the contribution of PGE_2_ to hypernociception following plantar incision does not seem to involve the upregulation of TTX-R VGSCs since the pharmacological inhibition of COX did not affect the expression of *Na_v_1.8* and *Na_v_1.9* in the DRG. Therefore, while PGE_2_ contributes to the onset of hypernociception, other mediators must be involved in the late stages of maintenance of nociceptive sensitization.

In addition to *COX-2*/PGE_2_, plantar incision also increased TNF-α levels both in the DRG and in plantar skin of mice. TNF-α is a pro-inflammatory and hypernociceptive cytokine produced by different cells and released during tissue damage [38]. In line with the present data, it has been shown that incisional wounds in mice are characterized by increased local levels of TNF-α [39,40]. Here, treatment with the anti-TNF-α monoclonal antibody infliximab markedly reduced hypernociception throughout the experimental period, suggesting a central role for this cytokine in nociceptive sensitization in this model. This result agrees with the many reports of TNF-α as an important mediator in the development and maintenance of painful conditions [41,42,43]. Differently from PGE_2_, TNF-α contributed to the upregulation of Na_v_1.8 and Na_v_1.9 mRNA in the DRG of incised mice, since the treatment with infliximab reduced the hyperexpression of these channels. Chen et al. [44] have shown that TNF-α increases the current density of both Na_v_1.8 and Na_v_1.9 in cultured DRG neurons. The authors also demonstrated that TNF-α levels are increased in the cerebrospinal fluid of neuropathic mice, leading to nociceptive sensitization by enhancing the current of Na_v_1.8 in the DRG [44]. Moreover, Fischer et al. [45] have shown the increased expression of Na_v_1.8 and Na_v_1.9 mRNA in the DRG of transgenic mice chronically exposed to TNF-α. Taken together, these data suggest that the pro-nociceptive effects of TNF-α in the mouse model of incisional pain are at least partially due to a positive modulation of Na_v_1.8 and Na_v_1.9.

TNF-α promotes transcriptional and post-translational alterations that culminate in increased TTX-R currents; both effects require the activation of intracellular MAPK pathways. MAPKs have many functions, including the modulation of neural plasticity, inflammation, and pain [23]. Among the members of the MAPK family, p38 has an important role in both peripheral and central nociceptive sensitization [46,47,48]. The activation of p38 by TNF-α, mainly through TNFR1 receptors, leads to two different outcomes: the phosphorylation of TTX-R sodium channels by p38, thereby increasing sodium influx into afferent nociceptive neurons [49,50], and the activation of the p38/NF-κB pathway, leading to the upregulation of TTX-R VGSCs and hypernociceptive mediators [20,51,52]. The present study evidenced that the pharmacological antagonism of p38 reduced plantar incision-induced hypernociception and downregulated the expression of Na_v_1.8 and Na_v_1.9 mRNA in the DRG of mice; the same effects were not observed when ERK and JNK were antagonized. These results indicate that p38 is an important element in the pathway leading to nociceptive sensitization mediated by TTX-R channels in the mouse model of incisional pain. Importantly, while COX-derived products seemed to be relevant in the acute hypernociceptive response, p38 seemed to be important for the persistence of hypernociception, since its inhibition by SB203580 only affected nociceptive thresholds from six hours onwards.

The transcription factor NF-κB is one of the many intracellular targets of p38. Upon activation in the cytoplasm, NF-κB migrates to the nucleus, where it modulates the expression of several genes involved in pain processing, including *TNF-α* and *COX-2* [20,23,52]. Accordingly, antagonizing NF-κB reduced both *TNF-α* and *COX-2*/PGE_2_ expression in the DRG and in the plantar skin of incised mice. The pharmacological antagonism of NF-κB also reduced the hyperexpression of *Na_v_1.8* and *Na_v_1.9* in the DRG, hence reducing the incision-induced hypernociception. Antagonizing NF-κB resulted in a pattern of responses that were very similar to those promoted by antagonizing p38. Both antagonists promoted antinociception with comparable kinetics and magnitude and reduced the expression of TTX-R VGSCs in very similar proportions, suggesting that the effects of p38 and NF-κB overlap in the same signaling pathway. In line with the present results, previous reports have demonstrated the up-regulatory effect of NF-κB on the expression of VGSCs in nociceptive neurons, including Na_v_1.7 [21,22] and Na_v_1.8 [53]. Therefore, is possible that the activation of NF-κB is the final step of the signaling cascade resulting in the upregulation of *Na_v_1.8* and *Na_v_1.9* in the mouse model of incisional pain.

Taken together, the present results, supported by data from the literature, subsidize the hypothesis for a mechanism by which nociceptive sensitization takes place in the mouse model of incisional pain. The early local production of PGE_2_ followed by upregulated expression of COX-2 in the DRG contributes to the initial process of nociceptive sensitization. TNF-α is also produced after plantar incision and perpetuates nociception for days, possibly by activating the p38/NF-κB pathway in the DRG, which results in the upregulation of *Na_v_1.8* and *Na_v_1.9*. Because NF-κB is also known to upregulate the expression of PGE_2_ and TNF-α, it can create a positive feedback loop that intensifies postoperative nociception.

Although the present work does not rule out the contribution of other pathways and mediators, it shows the activation of important signaling molecules that are relevant for incisional hypernociception by upregulating *Na_v_1.8* and *Na_v_1.9* in the DRG. These findings contribute to the understanding of postoperative pain and its pathophysiology, opening possibilities for the investigation of new pharmacological targets and treatments. Future studies must endeavor to determine additional components of this pathway, such as the cell types that are involved in the synthesis of TNF-α and PGE_2_, receptors activated by these hypernociceptive mediators during incision-induced nociceptive sensitization, as well as other effects of these mediators that have not been addressed herein.

## 4. Materials and Methods

### 4.1. Animals

Swiss mice (*Mus musculus*) were obtained from the Animal Facilities of the Gonçalo Moniz Institute (FIOCRUZ; Salvador, BA, Brazil) and kept in a room with controlled temperature (22–24 °C) and humidity (55–60%) and a 12:12 h light–dark cycle of artificial light. Mice were housed in micro-isolator cages with polycarbonate igloos as environmental enrichment and with free access to food and water. Inclusion criteria were sex (male mice) and body weight (22–28 g). Female mice were not used in this study because of the influence of hormonal fluctuations throughout the estrous cycle on the perception of painful stimuli. Groups of mice were formed by cluster random sampling, with two cages housing three mice each being randomly selected and assigned to a group (*n* = 6). Group size was determined based on previous studies that employed the same model [54,55]. Behavioral experiments were performed by researchers working in pairs. While one of them was responsible for handling mice and administering drugs, behavioral data was acquired by a blind evaluator. If a mouse displayed signs of extreme pain or distress or any noticeable behavioral alteration it would be discontinued from the experiment and euthanized—during the entire study, there was no need to force this endpoint. All procedures were reviewed and approved by the Ethics Committee for Animal Experimentation of Federal University of Bahia (CEUA/EMEVZ-UFBA; reference number 095/2018) and conducted in accordance with ethical principles established by the International Association for the Study of Pain [56].

### 4.2. Mouse Model of Incisional Pain

Plantar incision for mimicking postoperative pain was performed as described by Pogatzki and Raja [6] with minor modifications. Mice were anesthetized with isoflurane (Cristália Pharmaceutical Chemicals, Itapira, SP, Brazil) (2% for induction, 1.5% for maintenance). The plantar surface of the right hind paw was disinfected with 10% povidone-iodine (Rioquímica, São José do Rio Preto, SP, Brazil). Then, an 8-mm longitudinal incision was made in the hind paw with a #11 scalpel blade. The blade was initially positioned 2 mm away from the proximal edge of the heel and moved towards the toes, cutting skin and fascia in a single movement. The exposed muscle was elevated with tweezers, which were then moved back and forward three times to simulate surgical manipulation; muscle origin and insertion remained intact. The edges of the incision were brought together and gently pressed to allow hemostasis. The skin was apposed with two single sutures of 6-0 polypropylene. Mice were returned to their home cages and observed until full recovery from anesthesia. *Naïve* mice went through anesthesia and hind paw disinfection, but no surgical incision was made.

### 4.3. The von Frey Test for Mechanical Nociception

To access nociceptive sensitization caused by plantar incision, nociceptive thresholds to mechanical stimuli were measured with von Frey filaments (Stoelting; Chicago, IL, USA). Thresholds were evaluated daily for three days before surgery (baseline) and at different time points for up to six days after plantar incision. Mice were placed in acrylic cages upon a wired grid floor for thirty minutes to acclimate. The right hind paw was then touched with a series of filaments with logarithmically incremental stiffness (0.008–4 g) until they were slightly bent. Abrupt withdrawal of the touched paw was considered a positive response. The mechanical nociceptive threshold was calculated by the up-and-down method as described by Chaplan et al. [57] and represents the filament weight (g) to which mice respond in 50% of presentations.

### 4.4. Real-Time PCR

The expression of *Na_v_1.8* (*SCN10A*), *Na_v_1.9* (*SCN11A*), *TNF-α* (*TNF*), and *COX-2* (*Ptgs2*) genes was evaluated by Real-Time PCR in the DRG of mice. Total RNA was extracted from the DRG (L4-L5) with 1 mL of TRIzol reagent (Life Technologies, Grand Island, NY, USA) and its concentration was determined by photometric measurements. A High-Capacity cDNA Reverse Transcription Kit (Applied Biosystems, Foster City, CA, USA) was used in the synthesis of cDNA from 1 µg of RNA, according to the manufacturer’s recommendations. Synthesis of cDNA and RNA expression analysis was performed by Real-Time PCR using TaqMan Gene Expression Assay (Thermo Fisher Scientific, Waltham, MA USA) for *Na_v_1.8* (Mm00501467_m1), *Na_v_1.9* (Mm00449377_m1), *TNF-α* (Mm00443258_m1), and *COX-2* (Mm01307329_m1). A no-template control (NTC) and no-reverse transcription controls (No–RT) were included. All reactions were run in duplicate on an ABI7500 Sequence Detection System (Applied Biosystems) under standard thermal cycling conditions. The mean cycle threshold values from duplicate measurements were used to calculate the expression of the target gene. *Gapdh* (Mm99999915_g1) was used as a housekeeping gene. Gene expression was calculated using the formula 2^−ΔΔCt^ as previously described [58].

### 4.5. Na_v_1.8 and Na_v_1.9 Knock-Down

Knockdown for Na_v_1.8, Na_v_1.9, or both sodium channels in mice were produced by intrathecal antisense oligodeoxynucleotides (ODN) treatments. The antisense ODNs sequences 5′-TTG CCA TAA ACT TCT CTT C-3′ and 5′-TTC TCC TTG GCC TCT GTC T-3′ were directed against unique regions of the mouse mRNA for Na_v_1.8 (GenBank accession no. NM_009134.3), and Na_v_1.9 (GenBank accession no. NM_ 011887.3), respectively. Mismatch-ODN sequences 5′-TTC GCA TTA ACA TGT CAT C-3′ and 5′-TAC TGC TAG CCC TCA CTC T-3′ for Na_v_1.8 and Na_v_1.9, respectively, were derived from the antisense sequences by scrambling six bases (denoted by underline). Agreeing with the NCBI database on *Mus musculus,* there are no other homologous sequences. The 19-mer ODN were purchased lyophilized from Exxtend (Campinas, SP, Brazil), reconstituted in nuclease-free water with 0.9% NaCl to a concentration of 2 µg/µL, aliquoted and stored at −20 °C for use during treatments. Mice received intrathecal administrations of antisense or mismatch sequences (10 µg in 5 µL) once a day for four days (final dose of 40 µg) [17].

### 4.6. TNF-α Quantification by ELISA

Local TNF-α levels were estimated as described by Lima et al. [59]. Following euthanasia, the plantar skin from mice’s right hind paws was surgically removed 4 h, 1, 2, 4, or 6 days after plantar incisions. Tissue proteins were extracted following the proportion of 100 mg of tissue per mL of PBS, containing 0.4 M NaCl, 0.05% Tween 20, and protease inhibitors (0.1 mM PMSF, 0.1 mM benzethonium chloride, 10 mM EDTA, and 20 KIU aprotinin A/100 mL). Samples were centrifuged (3000 g for 10 min at 4 °C) and the supernatant was kept frozen at −80 °C until analysis. TNF-α levels were estimated using a commercially available immunoassay ELISA kit for mice (R&D System, Minneapolis, MN, USA), following the manufacturer’s instructions. Results were expressed as picograms of cytokine per milligram of protein.

### 4.7. Prostaglandin E_2_ Quantification

Local PGE_2_ levels were quantified as described by Pinheiro and Calixto [60]. Following euthanasia, the plantar skin from mice’s right hind paws was surgically removed 4 h, 1, 2, 4, or 6 days after plantar incisions. Skin samples were dipped in 2 mL of PBS containing heparin (5 IU/mL) and the COX inhibitor indomethacin (50 mg/mL) (Sigma, Saint Louis, MA, USA). Samples were homogenized with a Polytron homogenizer, centrifuged (1300 g for 10 min at 4 °C), and the supernatant was kept frozen at −80 °C until analysis. PGE_2_ levels were determined using a commercially available enzyme immunoassay kit (Cayman Chemical, Ann Arbor, MI, USA), following the manufacturer’s recommendations.

### 4.8. Pharmacological Assays

Pharmacological assays with antagonists and inhibitors were performed to investigate possible mechanisms that are relevant to nociceptive sensitization in the mouse model of incisional pain. Treatments included: anti-TNF-α monoclonal antibody infliximab (10 mg/kg, intraperitoneally) [61], non-selective COX inhibitor indomethacin (10 mg/kg, orally) [62], NF-kB antagonist PDTC (25–100 mg/kg, intraperitoneally) [63], p38 antagonist SB203580 (2.5–20 mg/kg, intraperitoneally) [64], ERK antagonist PD98059 (0.3–3 mg/kg, subcutaneously) [65], and JNK antagonist SP600125 (25–100 mg/kg, subcutaneously) [66]. Infliximab, indomethacin, and PDTC were purchased from Sigma (Saint Louis, MA, USA). SB203580, PD98059, and SP600125 were purchased from Tocris Bioscience (Bristol, UK). Treatments were made one hour before plantar incision and followed by nociceptive threshold evaluation and Real-Time PCR for detecting Na_v_1.8 and Na_v_1.9 transcription in the DRG, as described in previous sections.

### 4.9. Statistical Analyses

Data were presented as means ± SD of measurements made on six mice per group. Comparisons between three or more groups were made using one-way ANOVA with Tukey’s post-hoc test. For repeated measures, comparisons between groups were made by two-way ANOVA with Bonferroni’s post-hoc test. The factors analyzed were treatments, time, and treatment–time interaction. Data were analyzed using GraphPad Prism 8 computer software (GraphPad, San Diego, CA, USA). Statistical differences were considered to be significant at *p* < 0.05.

## Data Availability

Not applicable.
